# Interfacial Engineering of Iodine‐Polymer‐Carbon Hybrid Cathodes for High‐Energy Zn//I_2_ Microbatteries

**DOI:** 10.1002/advs.76819

**Published:** 2026-08-11

**Authors:** Yujia Fan, Sanat Nalini Paltasingh, Nibagani Naresh, Ruixiang Li, Kewei Chen, Monojit Mondal, Iman Pinnock, Xiaopeng Liu, Mingqing Wang, Saroj Kumar Nayak, Buddha Deka Boruah

**Affiliations:** ^1^ Institute For Materials Discovery University College London (UCL) London England UK; ^2^ School of Basic Sciences Indian Institute of Technology Bhubaneswar Khorda Odisha India; ^3^ School of Engineering and Materials Science Queen Mary University of London London England UK

**Keywords:** cathode‐confined iodine, microplotter printing, on‐chip energy storage, polyaniline, zinc//iodine microbattery

## Abstract

Miniaturized energy‐storage devices with high energy density, fast charge‐storage kinetics, and long‐term durability are essential for next‐generation on‐chip electronics. Among various candidates, zinc‐iodine (Zn//I_2_) microbatteries (MBs) are attractive owing to their intrinsic safety, low cost, and rapid iodine redox chemistry. Herein, a printed coplanar Zn//I_2_ MB is developed using a cathode‐confined iodine strategy, in which redox‐active iodine is immobilized within a rationally engineered polyaniline (PANI) and activated‐carbon (AC) (I_2_@AC‐PANI) composite cathode framework. The synergistic AC‐PANI architecture enhances iodine utilization, charge‐transfer kinetics, and electrochemical reversibility, resulting in improved capacity, rate capability, and cycling stability. Through a combination of ex situ spectroscopic analyses, electrochemical measurements, self‐discharge studies, and density functional theory (DFT) calculations, stronger interactions between iodine species and the AC‐PANI framework are identified, together with enhanced charge‐transfer characteristics compared with AC alone. As a result, the coplanar Zn//I_2_ microbattery delivers an areal energy of 36.35 µWh cm^−2^ at an areal power of 50 µW cm^−2^ and retains 86.4% of its initial capacity after 2000 cycles. This work demonstrates that cathode‐confined halogen chemistry combined with AC‐PANI engineering provides an effective strategy for developing high‐performance coplanar microbatteries and offers a promising route toward safe, integrable, and durable microscale energy‐storage systems.

## Graphical abstract

A cathode‐confined iodine strategy employing a synergistic polyaniline/activated‐carbon hybrid enables highly reversible halogen redox chemistry in coplanar Zn//I_2_ microbatteries. Enhanced iodine utilization, suppressed iodine dissolution, and accelerated charge‐transfer kinetics deliver durable, safe, and integrable microscale energy‐storage devices with competitive energy density and improved long‐term cycling stability.

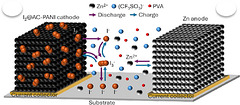

## 1 Introduction

The rapid development of miniaturized and integrated electronic systems, such as wearable sensors, implantable medical devices, and system‐on‐chip platforms, has created an urgent demand for micro‐scale energy storage devices that can simultaneously deliver high energy density, fast power response, and long‐term operational stability within a limited footprint [1]. Among various candidates, coplanar MBs, in which both the anode and cathode are patterned on the same substrate, have attracted increasing attention owing to their compatibility with on‐chip integration, scalable manufacturing, and flexible device design [2]. However, achieving high areal energy density in MBs remains a major challenge, especially under strict constraints on device footprint. Furthermore, emerging applications such as on‐chip wearable sensors and implantable medical devices require not only higher energy storage but also safer and more cost‐effective energy systems, demands that conventional Li‐ion and Na‐ion battery chemistries struggle to fully satisfy [3]. Aqueous Zn‐based MBs have emerged as promising alternatives to conventional Li‐based microsystems owing to their intrinsic safety, low cost, and environmental benignity [4]. In particular, the Zn//I_2_ MB system offers fast redox kinetics based on reversible iodine chemistry [5], making it attractive for miniaturized energy storage applications. Nevertheless, the practical implementation of Zn//I_2_ MBs is hindered by several intrinsic challenges, including iodine dissolution and shuttle effects, the formation of soluble polyiodide species, and sluggish redox reversibility [6] under confined micro‐scale conditions. These issues often result in rapid capacity fading, poor rate capability, and limited cycling stability, particularly in coplanar configurations with high surface‐to‐volume ratios [4].

To mitigate iodine‐related instability, various host materials such as porous carbons [7], polymers [8], and metal–organic frameworks [9] have been explored to physically confine iodine or chemically anchor polyiodide species. While porous carbon matrices can accommodate iodine through capillary effects, the interaction between carbon and iodine is typically weak [10], resulting in incomplete confinement and persistent shuttle phenomena. Conductive polymers, particularly PANI, have recently gained attention due to their tunable electronic structure, high electrical conductivity, and strong chemical affinity toward iodine species [11]. The *π*‐conjugated backbone and nitrogen‐containing functional groups of PANI enable *π*–*π* interactions and electrostatic stabilization with iodine, offering a potential route to suppress iodine dissolution and promote reversible redox conversion [12]. However, the integration of iodine‐active polymer frameworks into Zn//I_2_ MBs architectures, together with scalable fabrication and comprehensive mechanistic understanding, remains largely unexplored.

Herein, we report a Zn//I_2_ microbattery employing an I_2_@AC‐PANI composite cathode, in which redox‐active iodine is hosted within a AC‐PANI framework. The porous AC provides efficient iodine dispersion and electrolyte accessibility, while PANI serves as a conductive and iodine‐affinitive component that promotes charge transfer and strengthens interactions with iodine species. DFT calculations reveal that the AC‐PANI framework exhibits stronger interactions with I^−^, I_2_, and I_3_^−^ species than AC alone, suggesting enhanced iodine retention and improved interfacial charge‐transfer characteristics. Combined with characterisation and electrochemical analyses, the results indicate that the incorporation of PANI influences iodine redox behaviour and improves electrochemical reversibility. The effectiveness of the composite cathode is further demonstrated in a coplanar Zn//I_2_ microbattery, providing insight into the role of AC‐PANI hybrid hosts in iodine‐based energy‐storage systems and offering a practical approach for miniaturized aqueous microbatteries.

## 2 Results and Discussion

**FIGURE 1:**
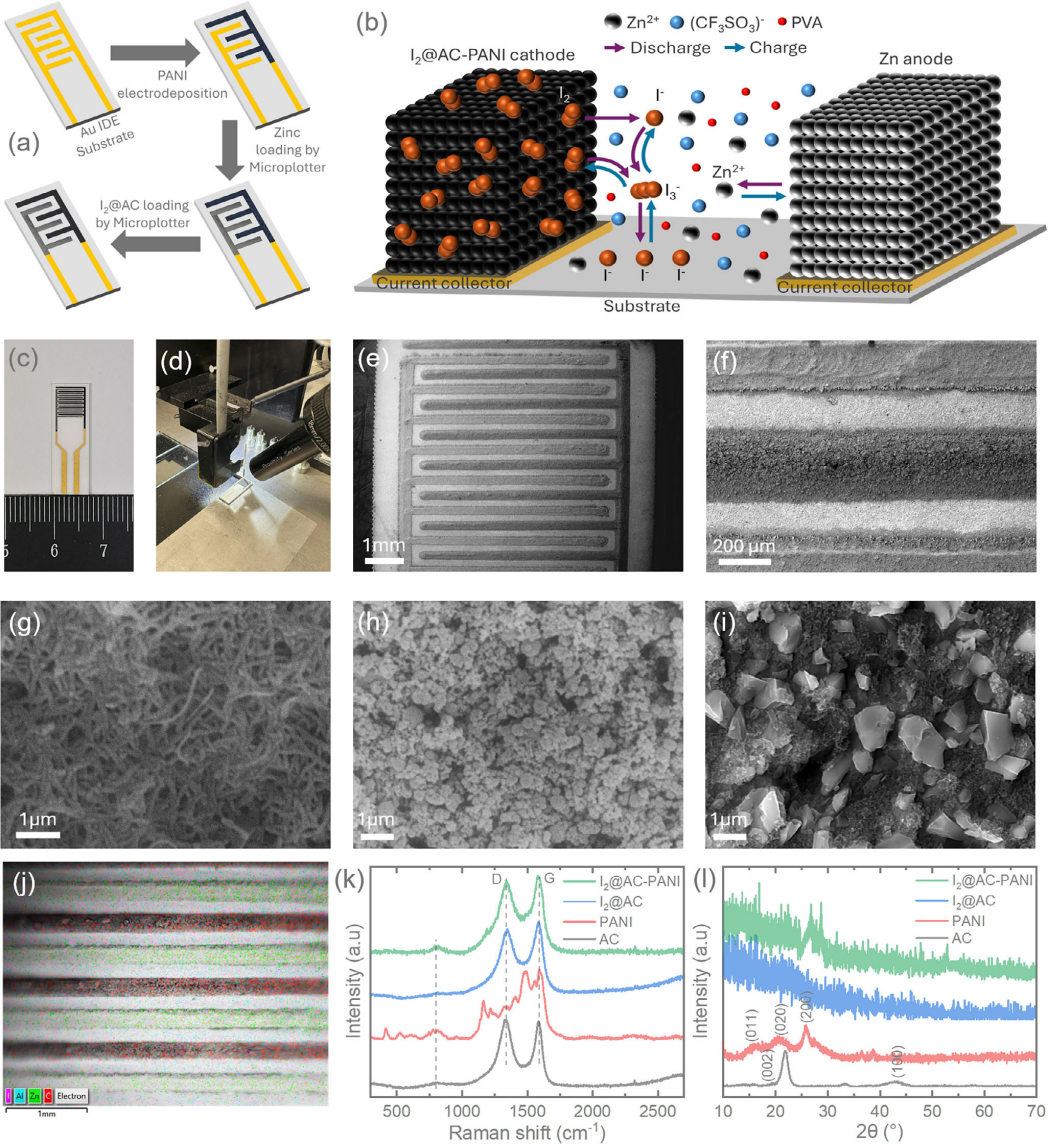
(a) Schematic illustration of the fabrication process for the coplanar Zn//I_2_ MB, showing sequential loading active materials onto of the Au IDEs. (b) Schematic working mechanism of the Zn//I_2_ MB with I_2_@AC‐PANI composite cathode and Zn anode in 3 m Zn(CF_3_SO_3_)_2_ electrolyte, highlighting the reversible I^−^/I_2_ redox conversion and Zn^2+^/Zn interphase reactions. (c) Digital photograph of the as‐fabricated Zn//I_2_ MB. (d) Digital image of the microplotter during electrode printing, illustrating precise ink deposition on the patterned substrate. (e) Low and (f) high‐magnification SEM image of the Zn//I_2_ MB. (g–i) SEM images of the raw electrode materials: (g) PANI, (h) Zn particles, and (i) AC. (j) EDX elemental mapping of the Zn//I_2_ MB, demonstrating homogeneous distribution of respective elements across electrodes. (k) Raman spectra of AC, PANI, I_2_@AC, and I_2_@AC‐PANI. (l) XRD patterns of AC, PANI, I_2_@AC, and I_2_@AC‐PANI.

The overall fabrication process of the coplanar Zn//I_2_ MBs is illustrated in Figure 1a. PANI was first electrodeposited onto a commercial gold interdigitated electrode (IDE), followed by sequential microplotter printing of Zn anode and I_2_@AC cathode inks to construct the Zn//I_2_ MBs. The working mechanism is schematically shown in Figure 1b, where the I_2_@AC‐PANI composite serves as a redox‐active cathode capable of reversible I^−^ ↔ I_2_ conversion, while the Zn anode undergoes a Zn^2+^/Zn interfacial reaction in a 3 m Zn(CF_3_SO_3_) _2_ electrolyte. The synergistic integration of the conductive PANI matrix, AC framework, and redox‐active iodine facilitates rapid ion/electron transport while effectively suppressing iodine shuttling within the confined cathode network (see discussion below). Figure 1c,d present a digital photograph of the fabricated Zn//I_2_ MB and the microplotter used for electrode printing, demonstrating the precision and reproducibility of the fabrication process. SEM images (Figure 1e,f) reveal a uniform and well‐defined interdigitated electrode pattern with consistent line width and spacing. The morphologies of the pristine materials are shown in Figure 1g–i. PANI nanofibers form a highly entangled network that provides abundant active sites and efficient electron transport pathways (Figure 1g). The Zn powder (Figure 1h) consists of nanoscale particles that promote high surface reactivity during cycling. The morphology of AC particles is shown in Figure 1i. Furthermore, EDS elemental mapping (Figure 1j) confirms the uniform distribution of iodine and carbon on the cathode fingers, while zinc is localized on the anode fingers of the IDE, verifying the successful spatial separation of the electrode materials.

Raman spectra (Figure 1k) show the characteristic D (∼1350 cm^−1^) and G (∼1580 cm^−1^) bands of carbon for both I_2_@AC and I_2_@AC‐PANI, with the latter exhibiting a slightly higher D‐band intensity (larger I_D_/I_G_), indicative of increased structural disorder and defect density introduced by PANI and iodine incorporation. The PANI‐related vibrations (C═N, C─N, and quinoid/benzenoid modes) are largely overlapped by the dominant carbon background but remain consistent with its partially doped emeraldine salt structure. The XRD patterns (Figure 1l) further verify the composite structure. PANI exhibits diffraction peaks at 16.2° (011), 20.6° (020), and 25.9° (200), characteristic of its semi‐crystalline nature [13]. Notably, no distinct crystalline I_2_ reflections are observed in the I_2_@AC pattern despite a nominal loading of 50 wt.%. This absence can be attributed to pore‐confinement‐induced amorphization or micro‐crystallization of iodine within the microporous carbon matrix, strong overlap with the broad carbon (002) halo around 20 – 30°, and partial charge‐transfer complexation that disrupts long‐range ordering. Consequently, iodine predominantly exists in highly dispersed and/or polyiodide forms that are invisible to XRD. The existence and confinement of iodine are further confirmed by XPS and UV–Vis analyses presented in the later section.

**FIGURE 2:**
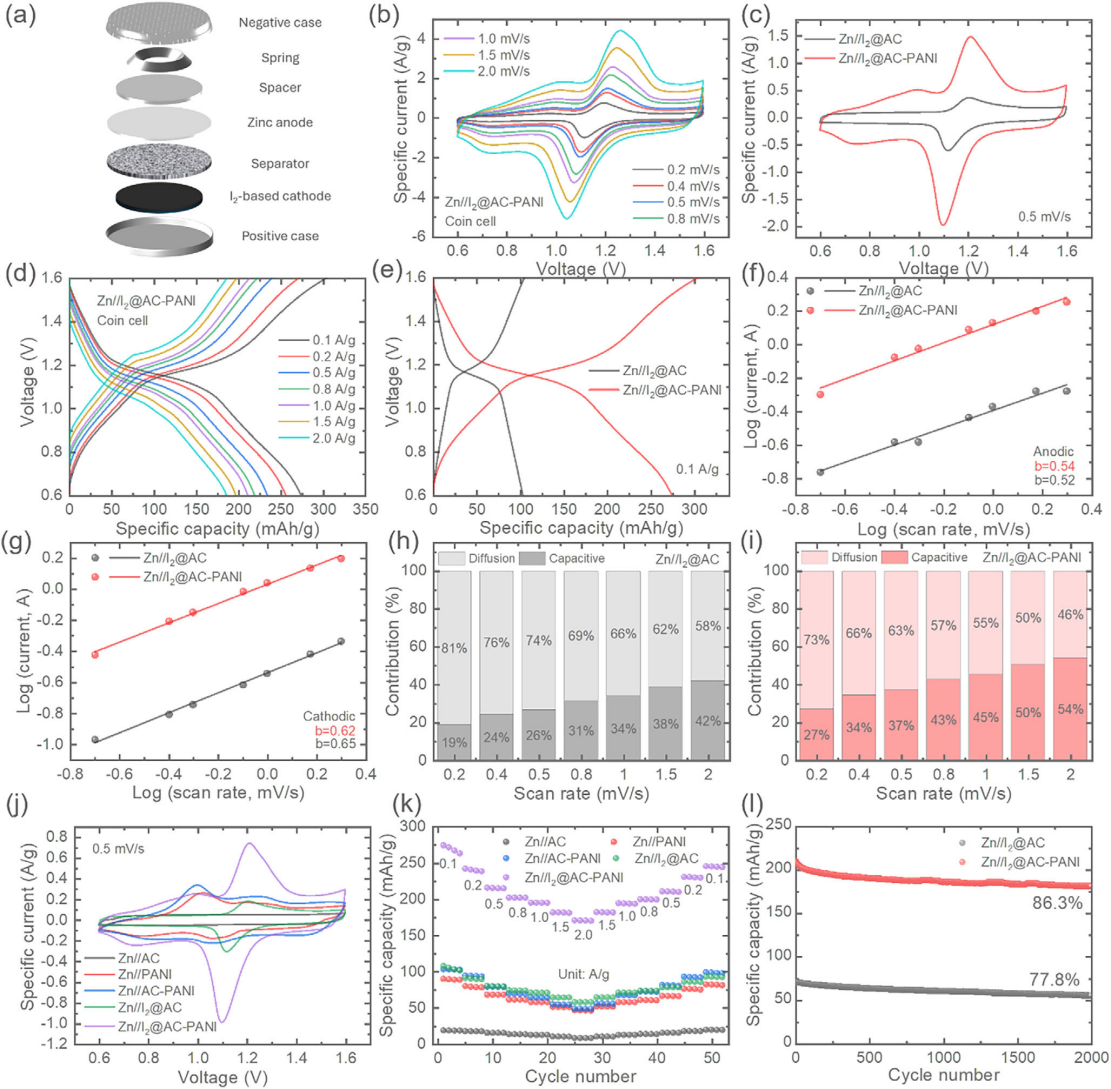
(a) Schematic illustration of the coin‐cell assembly. (b) CV curves of Zn//I_2_@AC‐PANI at scan rates ranging from 0.2 to 2.0 mV s^−1^. (c) CV comparison of Zn//I_2_@AC and Zn//I_2_@AC‐PANI at 0.5 mV s^−1^. (d) GCD profiles of Zn//I_2_@AC‐PANI under specific currents from 0.1 to 2.0 A g^−1^. (e) Comparison of GCD curves between Zn//I_2_@AC and Zn//I_2_@AC‐PANI at 0.1 A g^−1^. Determination of the (f) anodic and (g) cathodic b values for Zn//I_2_@AC and Zn//I_2_@AC‐PANI based on the relationship between peak current and scan rate. Capacitive and diffusion‐controlled charge contribution ratios as a function of scan rate for (h) Zn//I_2_@AC and (i) Zn//I_2_@AC‐PANI, respectively. (j) CV comparison of Zn//AC, Zn//PANI, Zn//AC‐PANI, Zn//I_2_@AC and Zn//I_2_@AC‐PANI at 0.5 mV s^−1^. (k) Rate performance of Zn//AC, Zn//PANI, Zn//AC‐PANI, Zn//I_2_@AC and Zn//I_2_@AC‐PANI. (l) Long‐term cycling stability of Zn//I_2_@AC and Zn//I_2_@AC‐PANI coin cells under specific current 1.0 A g^−1^.

Before evaluating the performance of the MBs with different cathode compositions, the cathode materials were first optimized through a series of electrochemical tests in coin cells. Figure 2a schematically illustrates the configuration of the Zn//I_2_ coin cells. As an initial step, the optimal I_2_ loading in AC was systematically investigated. Figure S1 compares the cyclic voltammograms (CV) of I_2_@AC cathodes with I_2_ contents ranging from 10 to 70 wt.%. At a low scan rate of 0.5 mV s^−1^, the integrated CV area increases progressively with increasing I_2_ loading, indicating that a larger fraction of I_2_ becomes electrochemically accessible when sufficient time is available for ion diffusion and redox conversion. In contrast, at a higher scan rate of 2.0 mV s^−1^, the CV area increases from 10 to 50 wt.% but slightly decreases at 70 wt.%, suggesting the onset of kinetic limitations at excessive iodine loading. Notably, the I_2_@AC electrode with 50 wt.% iodine exhibits the largest CV area at high scan rate and maintains an almost constant redox potential (∼1.1 V) across different scan rates, indicating highly reversible and kinetically favourable I^−^/I_2_ conversion. In contrast, the diminished high‐rate response at 70 wt.% suggests that a fraction of the excess I_2_ becomes electrochemically inactive, likely due to pore saturation, I_2_ aggregation, or limited electronic and ionic transport. These results identify 50 wt.% I_2_ as the optimal loading, balancing active material utilization, dispersion, and reaction kinetics. The corresponding CV and galvanostatic charge–discharge (GCD) profiles of the Zn//I_2_@AC (50 wt.%) coin cell are presented in Figures S1 and S2. Figure 2b presents the CV curves of the Zn//I_2_@AC‐PANI coin cell recorded at scan rates ranging from 0.2 to 2.0 mV s^−1^. The electrochemical response can be regarded as a combination of three contributions: (1) electric double‐layer charge storage from AC, (2) pseudocapacitive type redox reactions associated with the PANI backbone, and (3) iodine conversion reactions involving the I^−^/I_2_ redox couple. For comparison, the complete CV responses of Zn//AC, Zn//PANI, Zn//AC‐PANI, and Zn//I_2_@AC, are provided in Figure S2, while the corresponding 0.2 mV s^−1^ CV comparison is shown in Figure S2e. The progressive evolution of the CV profiles clearly demonstrates that the incorporation of both iodine and PANI leads to substantially enhanced electrochemical activity. CV comparison at 0.5 mV s^−1^ between Zn//I_2_@AC and Zn//I_2_@AC‐PANI is shown in Figure 2c. Despite containing the same iodine loading, the I_2_@AC‐PANI electrode exhibits significantly larger peak currents and integrated CV areas than I_2_@AC, indicating enhanced charge‐storage capability and more rapid iodine redox conversion. The stronger redox response suggests that a larger fraction of iodine becomes electrochemically accessible in the AC‐PANI framework. This behaviour can be attributed to the strong affinity of PANI toward iodine species, where the conjugated backbone and nitrogen‐containing functional groups facilitate charge‐transfer interactions and stabilize iodine species within the host structure. The galvanostatic charge–discharge (GCD) curves of Zn//I_2_@AC‐PANI at different current densities are presented in Figure 2d. Well‐defined charge/discharge plateaus are observed over the entire current range, confirming the highly reversible iodine redox process. Figure 2e further compares the GCD profiles of Zn//I_2_@AC and Zn//I_2_@AC‐PANI at 0.1 A g^−1^. The I_2_@AC‐PANI electrode delivers a substantially larger discharge capacity (∼274 mAh g^−1^) than I_2_@AC (∼103 mAh g^−1^), consistent with the CV results.

To investigate the charge‐storage kinetics, the relationship between the peak current (*i*) and the scan rate (*v*) follows *i*  =  *av^b^* [14], where the exponent *b* characterizes the charge‐storage mechanism (*b* ∼ 0.5 for diffusion‐controlled and *b* ∼1 for surface capacitive processes). As shown in Figure 2f,g, the anodic/cathodic *b* values for I_2_@AC are 0.52/0.65, whereas those for I_2_@AC‐PANI are 0.54/0.62, while the lower *b*values indicate a quasi‐diffusion‐controlled reduction process. According to Dunn's model, the total current response in CV can be expressed as, *i*(*V*)  = *k*_1_ *v* + *k*_2_ *v* ^1/2^ [15], where *k*_1_ *v* corresponds to the capacitive (surface‐controlled) contribution and *k*_2_ *v* ^1/2^represents the diffusion‐controlled contribution. The capacitive fraction, calculated as *k*_1_ *v*/(*k*_1_ *v* + *k*_2_ *v* ^1/2^), increases from 19% to 42% for I_2_@AC and from 27% to 54% for I_2_@AC‐PANI as the scan rate increases from 0.2 to 2.0 mV s^−1^ (Figure 2h,i). The higher capacitive contribution of the composite electrode demonstrates that iodine incorporated into the AC‐PANI framework facilitates faster interfacial charge transfer and enhances the overall pseudocapacitive behaviour compared with iodine loaded onto the AC cathode alone.

Figure 2j,k compare the electrochemical responses of Zn//AC, Zn//PANI, Zn//AC‐PANI, Zn//I_2_@AC, and Zn//I_2_@AC‐PANI coin cells. As shown by the CV curves recorded at 0.5 mV s^−1^, Zn//AC exhibits only a weak capacitive response characteristic of electric double‐layer charge storage, whereas Zn//PANI and Zn//AC‐PANI display broader redox features associated with the pseudocapacitive type behaviour of PANI. Upon iodine incorporation, distinct iodine‐related redox peaks emerge, confirming the contribution of the I^−^/I_2_ conversion reaction. Among all electrodes, Zn//I_2_@AC‐PANI delivers the largest current response and integrated CV area, indicating substantially enhanced charge‐storage capability. The corresponding rate performances further reveal the individual roles of AC, PANI, and iodine. While PANI contributes significant pseudocapacitive‐type capacity, the capacity of AC‐PANI remains close to the combined contributions of AC and PANI, suggesting that the AC‐PANI host itself does not introduce any anomalous capacity enhancement. To distinguish the contribution of iodine from that of the host materials, the effective iodine‐related capacity was estimated by subtracting the capacities of AC and AC‐PANI from those of I_2_@AC and I_2_@AC‐PANI, respectively. At 0.1 A g^−1^, the effective iodine‐related capacity increases from 89.1 mAh g^−1^ for I_2_@AC to 171.4 mAh g^−1^ for I_2_@AC‐PANI, corresponding to an enhancement of 92.4%. More importantly, this enhancement becomes increasingly pronounced with increasing current density, reaching 93.4%, 110.5%, 118.4%, 123.7%, 134.6%, and 145.4% at 0.2, 0.5, 0.8, 1.0, 1.5, and 2.0 A g^−1^, respectively. Such behaviour indicates that the improved performance of I_2_@AC‐PANI cannot be attributed solely to the pseudocapacitive type contribution of PANI. Instead, the AC‐PANI framework significantly improves the electrochemical accessibility of iodine species, particularly under kinetically demanding conditions. The progressively larger enhancement observed at higher current densities suggests that PANI not only contributes additional charge storage but also promotes iodine utilization through enhanced electronic conductivity, accelerated interfacial charge‐transfer kinetics, and stronger interactions between iodine species and the host framework, thereby facilitating faster and more reversible iodine redox conversion. The long‐term cycling performances of Zn//I_2_@AC and Zn//I_2_@AC‐PANI are compared in Figure 2l. After 2000 cycles at 1.0 A g^−1^, the I_2_@AC‐PANI electrode retains 86.29% of its initial capacity, significantly higher than the 77.78% retention observed for I_2_@AC. Combined with the DFT, UV–Vis, and XPS results presented later, the improved cycling stability supports the role of PANI in stabilizing iodine species, suppressing iodine loss, and promoting more reversible iodine redox reactions during prolonged cycling.

**FIGURE 3:**
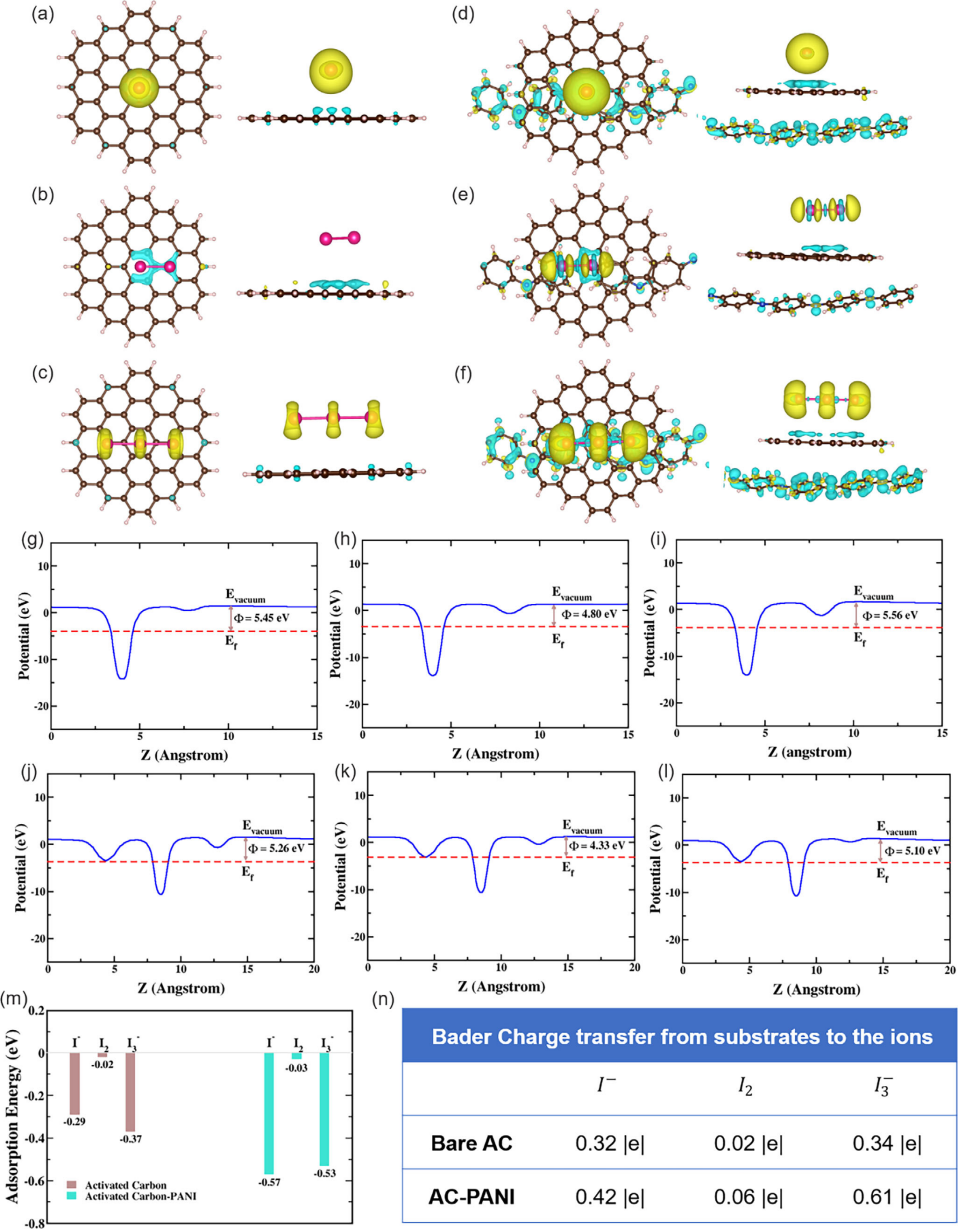
Charge density difference (CDD) plot for I^−^, I_2_, and I_3_^−^ adsorbed on AC (a–c) and AC‐PANI (d–f), respectively. The yellow colour represents the charge accumulation region, while the cyan colour indicates the charge depletion region, with an iso‐surface value of 0.0005 e/Å^3^. Work function plot of I^−^, I_2_, and I_3_^−^ adsorbed on AC (g–i) and on AC‐PANI (j–l), respectively. (m) The adsorption energies of the ionic species (I^−^, I_2_, and I_3_^−^) with both pristine AC and AC‐PANI demonstrate a comparatively stronger interaction between the ionic species and AC‐PANI. (n) Bader charge analysis showing the charge transfer from the host surface to the ionic species I^−^, I_2_, and I_3_^−^.

To gain a deeper understanding of the enhanced charge‐storage performance of the AC–PANI framework compared with pristine AC in halogen redox chemistry, DFT calculations were performed to elucidate the interactions of iodine species (I^−^, I_2_, and I_3_^−^) with bare AC and the AC‐PANI hybrid framework. The optimized geometries of the ionic species (I^−^, I_2_, and I_3_^−^) adsorbed on both AC and AC‐PANI are depicted in Figure S3. It should be noted that the present calculations were performed using a periodic PANI monolayer model with the same structural configuration throughout the study, enabling a consistent comparison between pristine AC and the AC‐PANI framework. Although different protonation or doping states of PANI may influence the absolute adsorption energies, the objective of this work is to establish the relative interactions of iodine species with the two host materials under an identical computational framework. Therefore, the conclusions are based on the comparative trends rather than the absolute adsorption energy values. As illustrated in Figure 3m,n, the adsorption energies of the ionic species (I^−^, I_2_, and I_3_^−^) on AC‐PANI are observed to be significantly greater than those on AC, which indicates that the incorporation of PANI markedly enhances the interaction between ionic species and activated carbon. As demonstrated in Figure 3a–f, the charge density difference (CDD) analysis reveals significant charge transfer from the host material, indicated by the charge depletion region (in cyan), to the adsorbates, represented by the charge accumulation region (in yellow). This observation confirms the anionic characteristics of the I^−^/I_3_^−^. It is noteworthy that AC‐PANI exhibits a more pronounced charge redistribution upon ionic adsorption in comparison to ACs. This assertion is further supported by the Bader charge analysis detailed in Figure 3n, which indicates that a greater quantity of charge is transferred from AC‐PANI to the I^−^/I_3_^−^ species than from the bare ACs. To further elucidate the electronic characteristics of these interactions, we evaluated the work function of ionic species adsorbed on both AC and AC‐PANI. As presented in Figure 3g–l, the adsorption of ionic species (I^−^, I_2_, and I_3_^−^) on AC‐PANI results in a lower work function compared to pristine ACs. This observation aligns with the enhanced electronegativity of the adsorbed species, as confirmed by Bader charge analysis. Collectively, these findings clearly indicate a stronger electronic coupling and superior adsorption affinity of AC‐PANI toward I^−^, I_2_, and I_3_^−^, establishing it as a more effective and efficient host compared to pristine AC.

**FIGURE 4:**
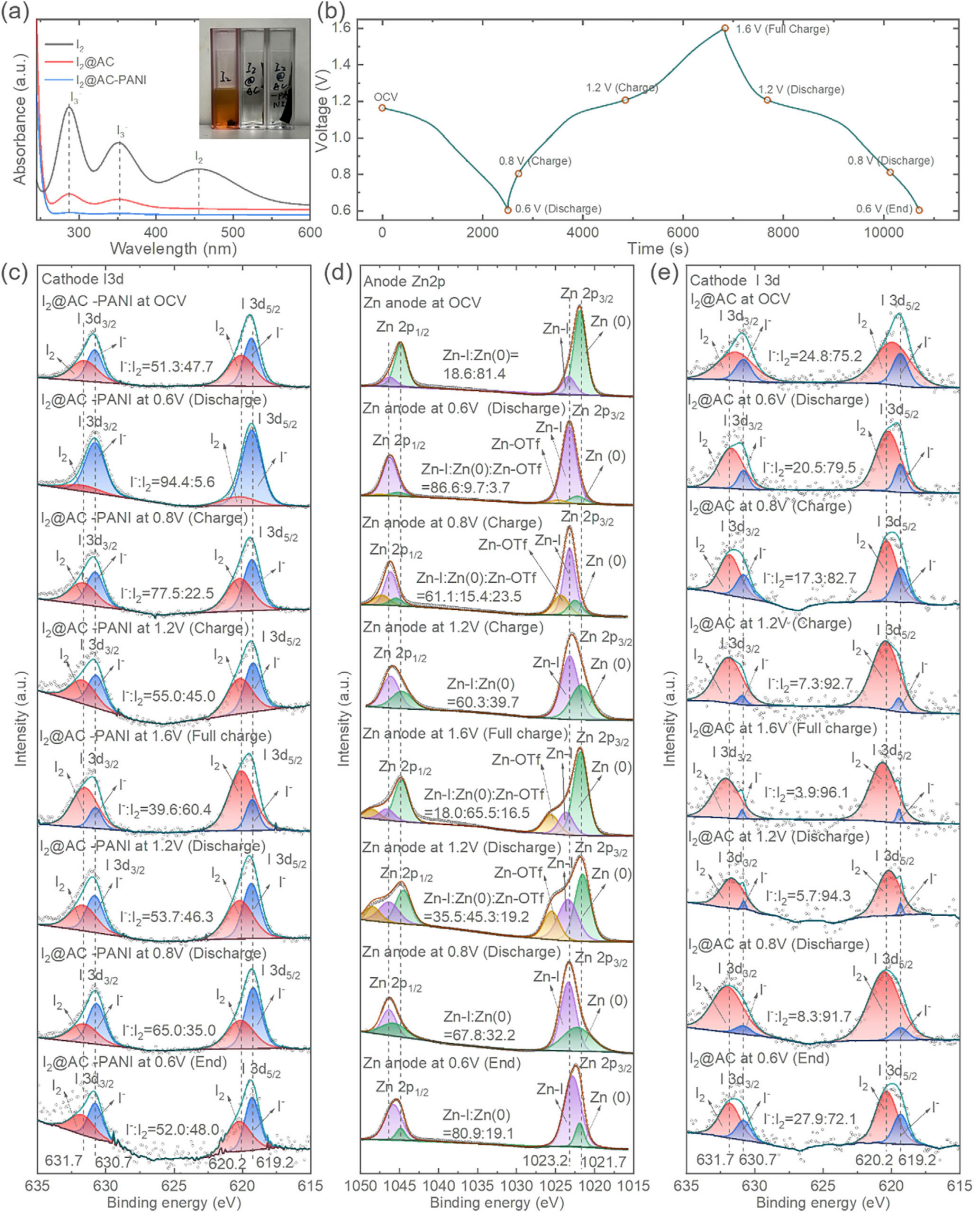
(a) UV–Vis spectra of I_2_, I_2_@AC, and I_2_@AC‐PANI after immersion in deionized water for 24 h. (b) GCD profile indicating the selected states for ex situ analysis. (c−e) Ex situ XPS spectra at different states of charge: (c) I 3d of the I_2_@AC‐PANI cathode, (d) Zn 2p of the Zn anode, and (e) I 3d of the I_2_@AC cathode.

The electrochemical charge‐storage behaviour and underlying reaction mechanisms of Zn//I_2_@AC and Zn//I_2_@AC‐PANI coin cells were systematically investigated at various charge/discharge states (Figure 4) using ex situ XPS, UV–Vis spectroscopy, and Raman spectroscopy analysis. To first evaluate the dissolution behaviour of polyiodide species in aqueous media, the cathodes (I_2_@AC and I_2_@AC‐PANI) as well as pristine iodine were immersed in deionized water for 24 h, followed by UV–Vis analysis of the corresponding solutions (Figure 4a). As expected, strong iodine‐related absorption bands corresponding to I_3_^−^ (∼288 and ∼353 nm) and molecular I_2_ (∼455 nm) [16] were observed when elemental iodine was immersed in water, indicating the spontaneous formation of polyiodide species. Similarly, the solution containing the I_2_@AC cathode exhibited clear I_3_^−^ signals, suggesting partial iodine dissolution through the formation of soluble polyiodides, which may contribute to active‐material loss during electrochemical cycling. In contrast, no obvious I_3_^−^ absorption bands were detected in the solution collected from the I_2_@AC‐PANI cathode after 24 h of immersion. These results suggest substantially reduced iodine dissolution and enhanced iodine retention within the PANI‐containing framework. To further elucidate the charge‐storage mechanism, the cathodes were harvested at different states of charge (Figure 4b) and analysed by ex situ XPS. In the pristine state (Figure S4), the I_2_@AC‐PANI cathode exhibits I^−^:I_2_:I−O area ratio of 23.8:65.0:11.2, whereas the I_2_@AC electrode shows a ratio of 21.0:75.0:4.0. The presence of I−O in the composite cathode suggests partial oxidation of iodine stabilized by the nitrogen‐containing and π‐conjugated sites of the PANI framework. In contrast, the pristine Zn anode displays only metallic Zn^0^, confirming a clean and stable interface prior to electrochemical operation. During charge and discharge (Figure 4c,e), the I_2_@AC‐PANI cathode exhibits a highly reversible I^−^ ↔ I_2_ conversion. The I^−^/I_2_ ratio evolves from 51.26:47.74 at open‐circuit voltage (OCV) to 94.4:5.6 at 0.6 V during discharge, followed by progressive iodine regeneration during charging (77.5:22.5 at 0.8 V → 55.0:45.0 at 1.2 V → 39.6:60.4 at 1.6 V). Upon the subsequent discharge process, the ratio returns to 53.7:46.3 (1.2 V) → 65.0:35.0 (0.8 V) → 52.0:48.0 (0.6 V), demonstrating a symmetric and highly reversible I^−^ ↔ I_2_ redox process with negligible polyiodide accumulation. In contrast, the I_2_@AC electrode remains predominantly iodine‐rich throughout the entire electrochemical process, with the I^−^/I_2_ ratio changing only slightly from 24.8:75.2 (OCV) to 20.5:79.5 (0.6 V discharge), followed by further iodine accumulation during charging (17.3:82.7 → 7.3:92.7 → 3.9:96.1), and remaining above 70% I_2_ even during discharge. This behaviour indicates sluggish I_2_ → I^−^ reduction and progressive iodine accumulation on the pristine AC surface, reflecting weaker iodine confinement and inferior redox reversibility. The Zn 2p spectra (Figure 4d) further reveal reversible interphase evolution at the Zn anode. The Zn−I/Zn^0^/Zn−OTf ratio evolves from 18.6/81.4/0% at OCV to 86.6/9.7/3.7% at 0.6 V during discharge, indicating the formation of Zn−I species. During charging, Zn^0^ is gradually regenerated, with ratios of 61.1/15.4/23.5% at 0.8 V and 60.3/39.7/0% at 1.2 V, eventually reaching 17.98/65.49/16.53% at 1.6 V (fully charged). Upon reverse discharge, the Zn−I fraction increases again (35.5/45.3/19.2% → 67.9/32.2/0% → 80.9/19.1/0% at 0.6 V), confirming a fully reversible Zn ↔ Zn−I interfacial conversion with only transient Zn−OTf species originating from the electrolyte. Collectively, these XPS results demonstrate that the PANI framework provides strong chemical anchoring for iodine, enabling a direct and highly reversible I^−^ ↔ I_2_ redox pathway. Meanwhile, the Zn anode undergoes a reversible Zn−I interphase formation and reduction with minimal parasitic by‐products. This behaviour is consistent with the UV–Vis and DFT results, which collectively indicate stronger interactions between iodine species and the AC‐PANI framework, enhanced iodine retention, and improved charge‐transfer characteristics compared with activated carbon alone. These features contribute to the superior reversibility and long‐term cycling stability of the I_2_@AC‐PANI system.

To further probe iodine dissolution during electrochemical operation, ex situ UV–Vis spectra of the electrolytes were recorded at different SoC (Figure S5a,b). Notably, no distinct absorption bands corresponding to I_3_^−^ (∼288 and ∼353 nm) or molecular I_2_ (∼455 nm) were detected in either system, and the overall absorbance remained nearly unchanged throughout the charging and discharging processes. The absence of characteristic iodine‐related signals suggests that the concentration of soluble iodine species in the 3 m Zn(CF_3_SO_3_)_2_ electrolyte is negligible, even at the fully charged state (1.6 V) during the first cycle. Further insight into the structural stability of the cathodes was obtained from ex situ Raman spectroscopy (Figure S5c,d). Both I_2_@AC and I_2_@AC‐PANI electrodes exhibit the characteristic D (∼1350 cm^−1^) and G (∼1580 cm^−1^) bands of carbon, which remain at nearly identical positions across different states of charge, confirming the structural robustness of the carbon framework. The intensity ratio (I_D_/I_G_) varies slightly between 1.05–1.10 for I_2_@AC and 1.10–1.15 for I_2_@AC‐PANI. The slightly higher I_D_/I_G_ ratio in the composite cathode suggests that the incorporation of PANI and iodine introduces mild structural disorder and additional defect sites, which are beneficial for iodine anchoring and interfacial charge transfer. Weak Raman bands below 1100 cm^−1^ are attributed to S─O_3_^−^ vibrations originating from trace CF_3_SO_3_^−^ residues of the electrolyte [17]. Meanwhile, characteristic PANI vibrational modes (C═N, C─N, and benzenoid/quinoid stretching) overlap with the dominant carbon and iodine features, making them difficult to distinguish. Overall, the preserved D/G band positions together with the slightly increased I_D_/I_G_ ratio in I_2_@AC‐PANI indicate a defect‐rich yet structurally stable carbon‐polymer framework that effectively confines iodine species. In addition, ex situ SEM images of both I_2_@AC and I_2_@AC‐PANI cathodes at different states of charge (Figure S6) show no discernible morphological changes. On the Zn anode, nanoscale flake‐like Zn deposits are observed, which are characteristic of Zn electrodeposition behaviour commonly reported in Zn‐based aqueous batteries.

**FIGURE 5:**
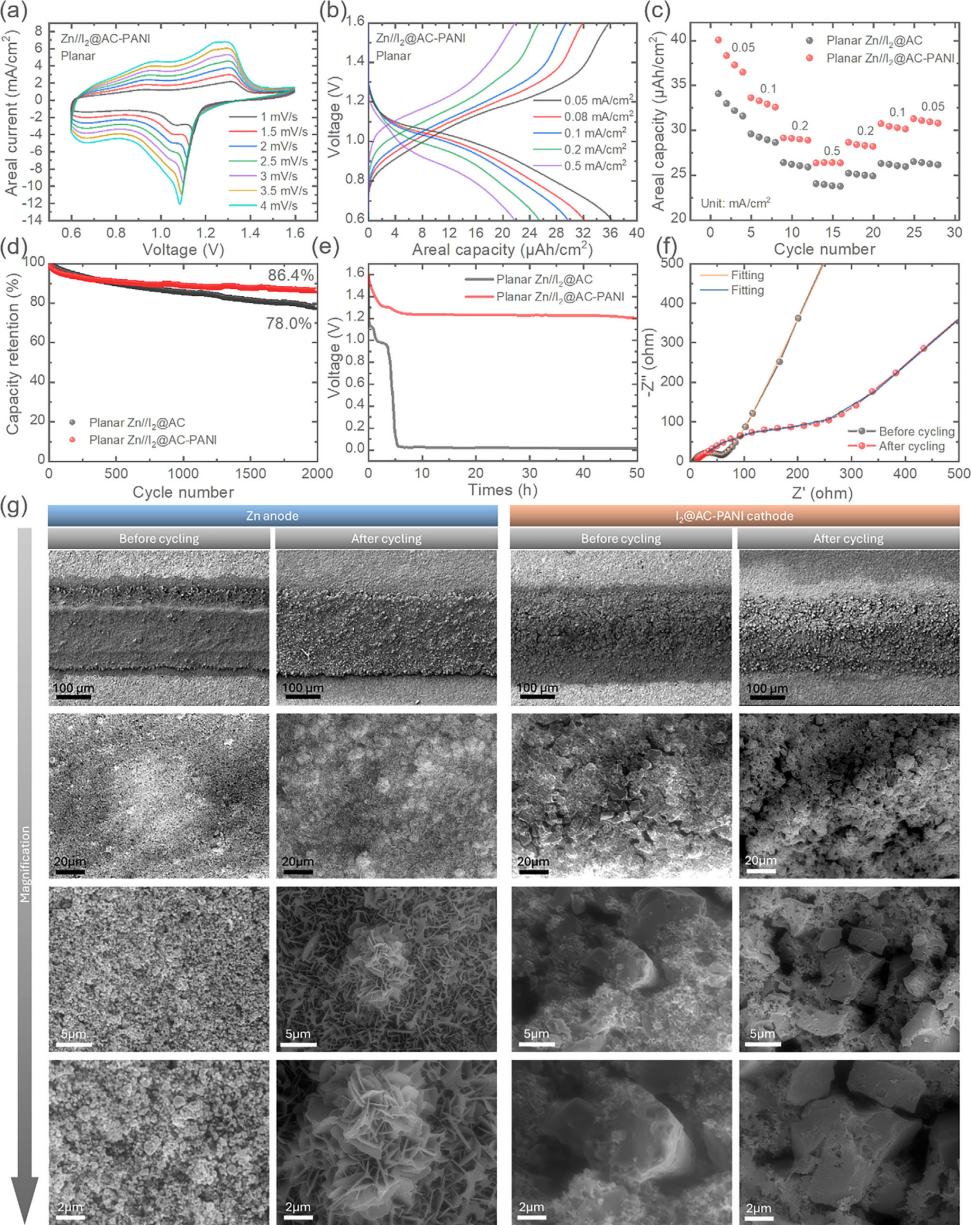
(a) CV curves and (b) GCD profiles of the planar Zn//I_2_@AC‐PANI MB. (c) Rate capability and (d) long‐term cycling stability of the MB devices. (e) Self‐discharge performance of MB devices. (f) Nyquist plots of the MBs before and after 250 cycling. (g) SEM images of the Zn anode and I_2_@AC‐PANI cathode before and after 250 cycling.

Subsequently, coplanar Zn//I_2_@AC‐PANI MBs (denoted as Zn//I_2_ MBs) were fabricated following the procedure described in Figure 1a, taking advantage of the synergistic iodine‐polymer‐carbon cathode architecture. The electrochemical performance of the devices is summarized in Figure 5. The CV curves of Zn//I_2_@AC‐PANI MB recorded at scan rates from 1.0 to 4.0 mV s^−1^ (Figure 5a) exhibit pronounced redox features dominated by the reversible I^−^/I_2_ conversion reaction, together with weaker contributions associated with the redox activity of the PANI framework. The major redox peaks located in the 1.0–1.3 V region can be attributed to iodine redox conversion, while the broader shoulder features originate from the electroactive PANI backbone. Importantly, the peak positions remain relatively stable with increasing scan rate and the overall CV profiles retain good symmetry, indicating favourable charge‐transfer kinetics and highly reversible electrochemical reactions within the composite electrode. Correspondingly, the GCD profiles shown in Figure 5b display distinct and reproducible voltage plateaus over a wide range of areal current densities, further confirming the reversible iodine redox chemistry and efficient charge‐storage behaviour of the I_2_@AC‐PANI MB. As shown in Figure 5c, the Zn//I_2_@AC‐PANI MB delivers areal capacities of 38.0, 33.1, 29.0, and 26.3 µAh cm^−2^ at areal currents of 0.05, 0.1, 0.2, and 0.5 mA cm^−2^, respectively. Even when the areal current is increased by an order of magnitude from 0.05 to 0.5 mA cm^−2^, the device retains approximately 69.2% of its initial capacity, demonstrating efficient ion/electron transport and favourable reaction kinetics within the I_2_@AC‐PANI framework. Moreover, when the areal current is returned to lower values, the capacity is largely recovered, indicating excellent reversibility and structural robustness of the printed electrode. Long‐term cycling stability was evaluated at 1.0 mA cm^−2^ (Figure 5d). The Zn//I_2_@AC‐PANI MB retains 86.4% of its initial capacity after 2000 cycles, significantly outperforming the Zn//I_2_@AC device, which retains only 78.0% under identical conditions. To further evaluate the ability of the host framework to retain electrochemically active iodine species, the self‐discharge behaviour of the devices was investigated after charging to 1.6 V (Figure 5e). Compared with Zn//I_2_@AC, the Zn//I_2_@AC‐PANI MB exhibits significantly improved voltage retention during open‐circuit storage. While the voltage of Zn//I_2_@AC decreases rapidly with time, the Zn//I_2_@AC‐PANI device maintains a substantially higher operating voltage throughout the monitoring period, indicating reduced self‐discharge. This improved voltage retention is consistent with the stronger interactions between iodine species and the AC‐PANI framework revealed by the DFT calculations, which favour the retention of electrochemically active iodine species within the cathode. In addition, the redox‐active PANI framework may provide supplementary charge‐storage and buffering effects that help stabilize the electrode potential during open‐circuit relaxation. Together, these factors contribute to the superior self‐discharge performance of the I_2_@AC‐PANI MB. The electrochemical impedance spectra before and after cycling are shown in Figure 5f. Although the overall impedance increases after prolonged cycling, the change remains relatively moderate, suggesting that the conductive pathways within the electrode are largely preserved. This observation agrees well with the stable cycling performance shown in Figure 5d. Further structural insights are provided by the SEM images in Figure 5g. After prolonged cycling, the Zn anode exhibits localized dendritic deposits associated with repeated Zn plating/stripping processes; however, no severe electrode fracture or short‐circuiting features are observed. More importantly, the I_2_@AC‐PANI cathode largely preserves its porous framework and structural integrity after cycling, without obvious delamination or structural collapse. The preserved morphology confirms the mechanical stability of the polymer‐carbon host and its ability to accommodate repeated iodine redox reactions. Overall, the combination of high areal capacity, excellent rate capability, low self‐discharge behaviour, and long‐term cycling stability demonstrates the effectiveness of the I_2_@AC‐PANI cathode design for coplanar Zn//I_2_ MBs. The synergistic effects of the conductive PANI network, AC, and enhanced iodine‐host interactions collectively contribute to improved iodine utilization, reaction reversibility, and durable electrochemical performance.

**FIGURE 6:**
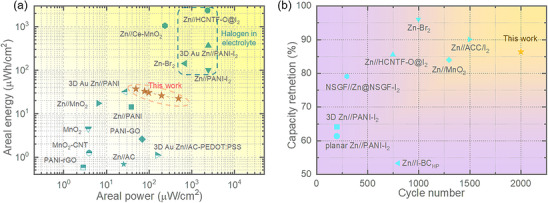
(a) Ragone plot comparing the areal energies and areal powers of our co‐planar MB with various published MBs: Zn//HCNTF‐O@I_2_ [18], 3D Au and planar Zn//PANI‐I_2_ [19], Zn‐Br_2_ [20], Zn//PANI [21], 3D Au Zn//PANI [22], screen printed Zn//MnO_2_ [23], and Zn//Ce‐MnO_2_ [24], PANI‐rGO [25], MnO_2_‐CNT [26], MnO_2_ [27], PANI/GO [28],Zn//AC [29], 3D Au Zn//AC‐PEDOT: PSS [30]. (b) Comparison of cycling stability with other halogen‐based Zn ion batteries, including Zn//HCNTF‐O@I_2_ [18], 3D Au and planar Zn//PANI‐I_2_ [19], Zn//Br_2_ [20], screen‐printed Zn//MnO_2_ [23], NSGF//Zn@NSGF‐I_2_ [31], Zn//ACC/I_2_ [32], Zn//I‐BC_HP_ [33].

Furthermore, the areal energy and power densities of the Zn//I_2_ MB were benchmarked against representative micro‐energy‐storage devices reported in the literature (Figure 6a). The device delivers an areal energy of 36.35 µWh cm^−2^ at an areal power of 50 µW cm^−2^ and maintains 21.84 µWh cm^−2^ at 500 µW cm^−2^, demonstrating a balanced energy‐power output. Although several Zn//Br_2_ and Zn//I_2_ microbatteries employing halogen‐active species in the electrolyte or complex three‐dimensional architectures achieve higher energy densities, the present device exhibits competitive performance using a simple printed coplanar configuration in which iodine is confined within the cathode. This design offers a practical route toward scalable and integrable micro‐energy‐storage systems. To further assess device durability, the cycling stability was compared with representative halogen‐based MBs reported in the literature (Figure 6b). The Zn//I_2_@AC‐PANI MB retains 86.4% of its initial capacity after 2000 cycles, placing it among the most durable reported planar halogen‐based MBs.

## 3 Conclusions

In summary, we have developed a printed coplanar Zn//I_2_ MB enabled by a rationally engineered I_2_@AC‐PANI composite cathode, achieving an improved balance between high areal energy density, favourable rate capability, and long‐term cycling stability within a compact on‐chip architecture. By integrating redox‐active iodine with a conductive and electrochemically active PANI framework supported by a porous activated‐carbon scaffold, a synergistic electrode architecture was established that promotes efficient iodine utilization and highly reversible iodine redox chemistry.

Comprehensive electrochemical characterization, UV–Vis spectroscopy, ex situ XPS analyses, self‐discharge measurements, and DFT calculations consistently demonstrate that the AC‐PANI framework exhibits stronger interactions with iodine species and enhanced charge‐transfer characteristics compared with activated carbon alone. As a result, the composite host improves the electrochemical accessibility of iodine species, enhances reaction reversibility, and contributes to improved cycling stability and voltage retention. Additional control experiments further reveal that the effective iodine‐related capacity in the AC‐PANI framework is substantially higher than that in the AC host, highlighting the important role of the polymer–carbon framework in improving iodine utilization. The resulting coplanar Zn//I_2_ microbattery delivers an areal energy density of up to 36.35 µWh cm^−2^ at an areal power density of 50 µW cm^−2^ and retains 86.4% of its initial capacity after 2000 cycles. These results demonstrate that the synergistic coupling of iodine chemistry, conductive polymer frameworks, and porous carbon hosts provides an effective strategy for developing high‐performance coplanar MBs. The scalable microplotter‐printing process and coplanar device architecture presented here offer a promising pathway toward safe, integrable, and high‐performance microscale energy‐storage systems for next‐generation wearable electronics, miniaturized devices, and system‐on‐chip applications.

## Author Contributions

**Kewei Chen**: data curation, writing – review and editing. **Buddha Deka Boruah**: supervision, resources, project administration, funding acquisition, writing – original draft, writing – review and editing, conceptualization. **Nibagani Naresh**: data curation, formal analysis, writing – original draft, writing – review and editing. **Yujia Fan**: conceptualization, methodology, data curation, formal analysis, validation, investigation, visualization, writing – original draft, writing – review and editing. **Monojit Mondal**: data curation, writing – review and editing. **Mingqing Wang**: writing – review and editing. **Xiaopeng Liu**: data curation, writing – review and editing. **Iman Pinnock**: data curation, writing – review and editing. **Ruixiang Li**: data curation, writing – review and editing. Sanat Nalini Paltasingh: data curation, formal analysis, writing – original draft, writing – review and editing. Saroj Kumar Nayak: supervision, resources, writing – review and editing.

## Conflicts of Interest

The authors declare no conflicts of interest.

## Supporting information




**Supporting File**: advs76819‐sup‐0001‐SuppMat.pdf.

## Data Availability

The data that support the findings of this study are available from the corresponding author upon reasonable request.
