# Supplementary material for: Interfacial Engineering of Iodine‐Polymer‐Carbon Hybrid Cathodes for High‐Energy Zn//I2 Microbatteries
Source: Adv Sci (Weinh). 2026 Aug 11:e76819. Online ahead of print. doi: 10.1002/advs.76819 (PMC13460406; doi:10.1002/advs.76819)
Supplement: Supplementary file 1 [file ADVS-9999-e76819-s001.pdf]

Support Information

For

Interfacial Engineering of Iodine-Polymer-Carbon Hybrid Cathodes for High-Energy Zn//I_2 Microbatteries

Yujia Fan[1], Sanat Nalini Paltasingh[2], Nibagani Naresh[1], Ruixiang Li[3], Kewei Chen[1], Monojit Mondal[1], Iman Pinnock[1], Xiaopeng Liu[1], Mingqing Wang[1], Saroj Kumar Nayak[2], Buddha Deka Boruah[1],*

[1]Institute for Materials Discovery, University College London (UCL), London WC1E 7JE, United Kingdom

[2]School of Basic Sciences, Indian Institute of Technology Bhubaneswar, Khorda, Odisha 752050, India

[3]School of Engineering and Materials Science, Queen Mary University of London, London E1 4NS, United Kingdom

Corresponding author: Dr. Buddha Deka Boruah

Email: b.boruah@ucl.ac.uk

Experimental Section

Materials: Sulfuric acid (H_2SO_4) and aniline (Sigma-Aldrich) were used for the electrodeposition of PANI. Zinc nanopowder (average particle size 40–60 nm) and triethylene glycol monomethyl ether (TEGMME) were also obtained from Sigma-Aldrich. Activated carbon and Super-P conductive carbon black were supplied by MTI Corporation, while iodine powder was purchased from Sigma-Aldrich. Poly(vinylidene fluoride) (PVDF) powder was obtained from Alfa Aesar, and the corresponding solvent, *N,N*-dimethylformamide (DMF), was sourced from Severn Biotech Ltd. All chemicals were used as received without further purification. For the gel electrolyte, poly(vinyl

alcohol) (PVA, Sigma-Aldrich) and zinc trifluoromethanesulfonate ($Zn(CF_3SO_3)_2$), Fluorochem) were employed.

Electrodeposition of PANI: Flat interdigitated gold electrodes (Au IDEs, DRP-IDEAU200, line/gap width 200 μm) were purchased from Metrohm U.K. Ltd. Prior to use, the IDEs were sequentially cleaned with distilled water and isopropanol, then dried under a nitrogen stream. The PANI electrodeposition electrolyte was prepared by dissolving 1 M H_2SO_4 (95-97%) and 0.5 M aniline in 50 mL of distilled water, followed by magnetic stirring for 3 h to obtain a homogeneous solution. A Pt wire and an Ag/AgCl electrode served as the counter and reference electrodes, respectively. PANI was electrodeposited onto one side of the Au IDE or onto cut graphene paper (for coin-cell testing) at a constant potential of 0.85 V for 20 s. After deposition, the electrodes were thoroughly rinsed with distilled water and dried in air.

Preparation of electrode inks: For the I_2@AC ink, 0.44 g of solid iodine was first dissolved in 5 g of triethylene glycol monomethyl ether (TEGMME) under ultrasonication for 30 min. Subsequently, 0.44 g of activated carbon powder was added to the iodine solution and further sonicated for 10 min to achieve uniform dispersion. Then, 0.10 g of Super-P conductive carbon black was introduced, followed by an additional 10 min of sonication. Finally, 0.20 g of a 10 wt% PVDF solution in DMF was added as a binder, and the mixture was sonicated for another 10 min to yield a well-dispersed I_2@AC ink.

For the Zn ink, 0.88 g of zinc powder, 0.10 g of Super-P, and 0.20 g of 10 wt% PVDF/DMF solution were combined and homogenized by dip-sonication until a uniform dispersion was obtained.

Preparation of gel electrolyte: Poly(vinyl alcohol) (PVA, 1 g) was dissolved in 10 mL of deionized water under continuous stirring at 85 °C until a clear solution was obtained. Subsequently, 10.9 g of zinc trifluoromethanesulfonate ($Zn(CF_3SO_3)_2$) was gradually added to the hot PVA solution under stirring to ensure complete dissolution and homogeneous mixing. The resulting viscous gel electrolyte was allowed to cool to room temperature before use.

Coin cell tests: Graphene paper was used as the current collector. The prepared electrode inks were drop-cast onto pristine graphene paper or PANI-coated graphene

paper, followed by drying at 40 °C overnight. The dried cathodes were then punched into 14 mm diameter discs.

Coin cells (CR2032 type) were assembled in the following sequence: the cathode was first placed in the center of the cell case, followed by a 19 mm diameter Whatman glass microfiber filter as the separator. Subsequently, 150 μL of 3 M $Zn(CF_3SO_3)_2$-PVA gel electrolyte was added dropwise onto the separator. A 14 mm diameter zinc foil (0.07 mm thick) was used as the anode and placed atop the separator, followed by a 1 mm thick spacer and a 1.1 mm thick spring. The assembly was then sealed and crimped under a pressure of 750 psi to complete the cell.

Electrochemical performance was evaluated using a Bio-Logic MPG-2 battery testing system. Cyclic voltammetry (CV) was performed within a voltage window of 0.6 - 1.6 V at scan rates ranging from 0.2 to 2.0 mV s^{-1}, while galvanostatic charge-discharge (GCD) tests were conducted at current densities from 0.1 to 2.0 A g^{-1}. Long-term cycling stability was evaluated at 1 A g^{-1} for 2000 cycles using a Neware battery tester.

Fabrication of Zn//I_2 MBs: The printing of coplanar Zn//I_2 MBs was performed using a SonoPlot Microplotter Proto equipped with a 20 μm nozzle. The interdigitated electrode (IDE) pattern for the Zn//I_2 MB was designed using SonoDraw software. During the printing process, the nozzle was carefully aligned with the starting point of the IDE pattern, and the feature size and printing mode were set to 50 μm and spray mode, respectively. The applied printing voltages were 8 V for the zinc electrode ink and 10 V for the I_2@AC electrode ink.

Material characterizations: The morphology and microstructure of the raw materials were examined using scanning electron microscopy (SEM, ZEISS EVO LS15). Raman spectroscopy was conducted on a RENISHAW inVia Raman microscope to obtain structural and bonding information of the powders. X-ray diffraction (XRD) measurements were performed using a Malvern Panalytical Aeris diffractometer to analyze the crystalline phases.

X-ray photoelectron spectroscopy (XPS) was carried out using a Thermo Scientific NEXSA system equipped with a monochromated Al Kα X-ray source, spherical sector analyzer, and multichannel resistive plate detector. Spectra were collected at 19.2 W with an X-ray beam size of 400 × 200 μm. Survey scans and high-resolution scans were acquired at pass energies of 200 eV and 40 eV, respectively. Electronic charge

neutralization was achieved using a dual-beam low-energy electron/ion source (Thermo Scientific FG-03). All spectra were recorded at a pressure below 1×10^{-8} Torr and a temperature of 294 K. Data processing was conducted using CasaXPS v2.3.20rev1.0o. UV-Vis spectroscopy (PerkinElmer) was employed to analyze the electrolyte and monitor iodine-related species during electrochemical cycling.

Electrochemical testing: During the assembly of the Zn//I_2 MBs, two copper foil strips were attached to the ends of the printed electrodes using silver paste to establish electrical contacts, and the junctions were insulated with Kapton tape. The assembled device was then placed into a Fisherbrand disposable cuvette (14955125), after which the 3M $Zn(CF_3SO_3)_2$ PVA gel electrolyte was injected to fully immerse the electrodes. The top of the cuvette was sealed with parafilm, and the exposed copper foil leads were fixed to the outer wall for external electrical connection.

Electrochemical measurements of the Zn//I_2 MBs were carried out using multiple testing systems. Cyclic voltammetry (CV) was performed at scan rates ranging from 1 to 4 mV s^{-1}, and galvanostatic charge-discharge (GCD) tests were conducted at areal current densities between 0.05 and 0.5 mA cm^{-2} using a Bio-Logic MPG-2 battery testing system. Long-term cycling stability was evaluated at 1 mA cm^{-2} for 2000 cycles using a Neware battery tester. Electrochemical impedance spectroscopy (EIS) was measured over the frequency range of 10 mHz–100 kHz with a voltage amplitude of 10 mV using an IVIUM Potentiostat/Galvanostat.

Density functional theory (DFT) modelling: All density functional theory (DFT)[1–3] calculations were performed using the vienna ab initio simulation package (VASP)[4,5]. The generalized gradient approximation[6] of Perdew-Burke-Ernzerhof[7] (GGA-PBE) was employed to characterize the exchange correlation potential. A plane-wave cutoff energy of 500 eV was considered for all calculations. All geometrical structures were optimised by fully relaxing atomic positions until the Hellmann-Feynman forces on each atom were reduced below a threshold of 0.01 eV, with a total energy convergence criterion of 1×10^{-5} eV. To include van der Waals interactions, the DFT-D3 method[8] was applied to correct for potential energy and interatomic forces. For structure optimization, the Brillouin zone was sampled using gamma-centered k-point grids of 3×3×1.

To investigate the interaction of ionic species with activated carbon and the composition of activated carbon situated atop polyaniline (PANI), we have employed a model of activated carbon consisting of a 19-ring graphene sheet terminated with hydrogen atoms (coronene: $C_{54}H_{18}$) along with a monolayer of PANI, in accordance with our previous research findings[9].

The adsorption energy is calculated using the following formula:

$$\mathbf{E_{ad} = E_{surface+adatom} - E_{surface} - E_{adatom}}$$

Where, $E_{surface+adatom}$, $E_{surface}$, and E_{adatom} correspond to the total energies of the system constituting adatom adsorbed on the surface, the energy of the clean surface, and the energy of the isolated adatom, respectively. According to the formula, a more negative value of binding energy will indicate a stronger interaction.

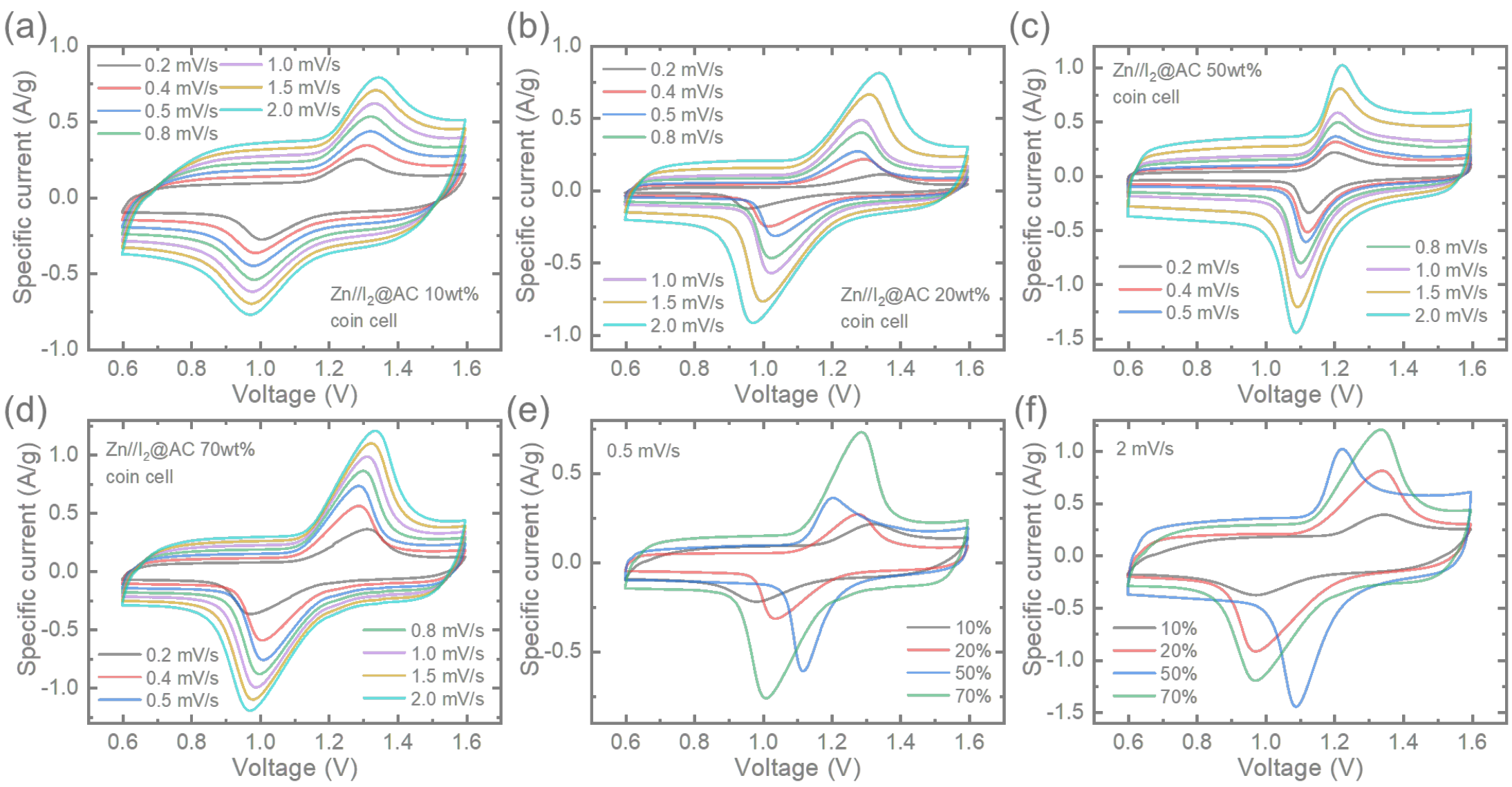

Figure S1. *CV curves of Zn//I_2@AC coin cells with different I_2 loadings: (a) 10 wt%, (b) 20 wt%, (c) 50 wt%, and (d) 70 wt%. (e, f) Comparison of CV profiles for Zn//I_2@AC with 10, 20, 50, and 70 wt% I_2 loadings at scan rates of 0.5 and 2.0 mV s^{-1}, respectively.*

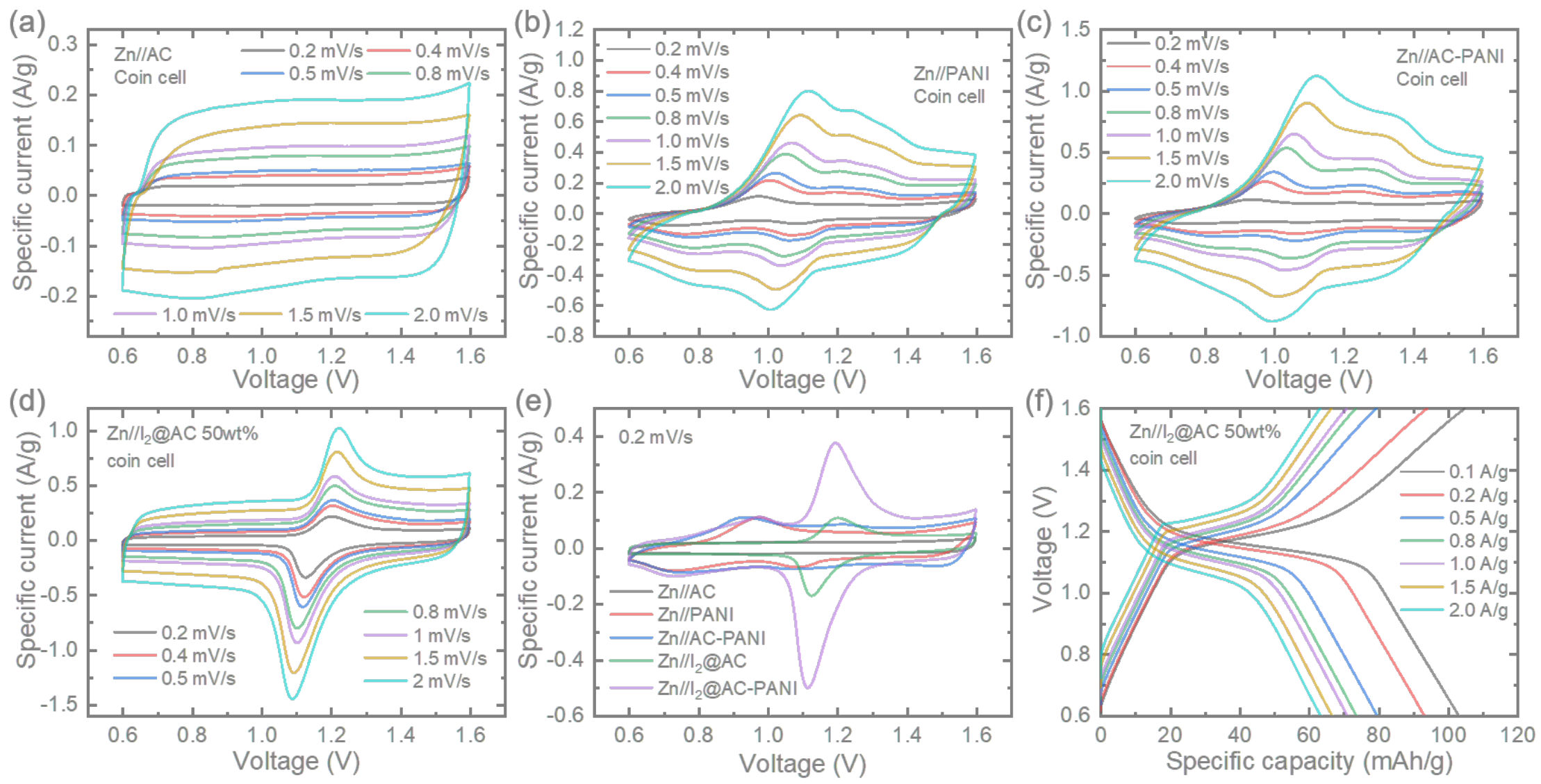

Figure S2. *CV curves of (a) Zn//AC, (b) Zn//PANI, (c) Zn//AC-PANI, (d) Zn//I$_2$@AC and (e) CV comparison at 0.2 mV s$^{-1.}$ (f) GCDs of Zn//I$_2$@AC at different specific currents.*

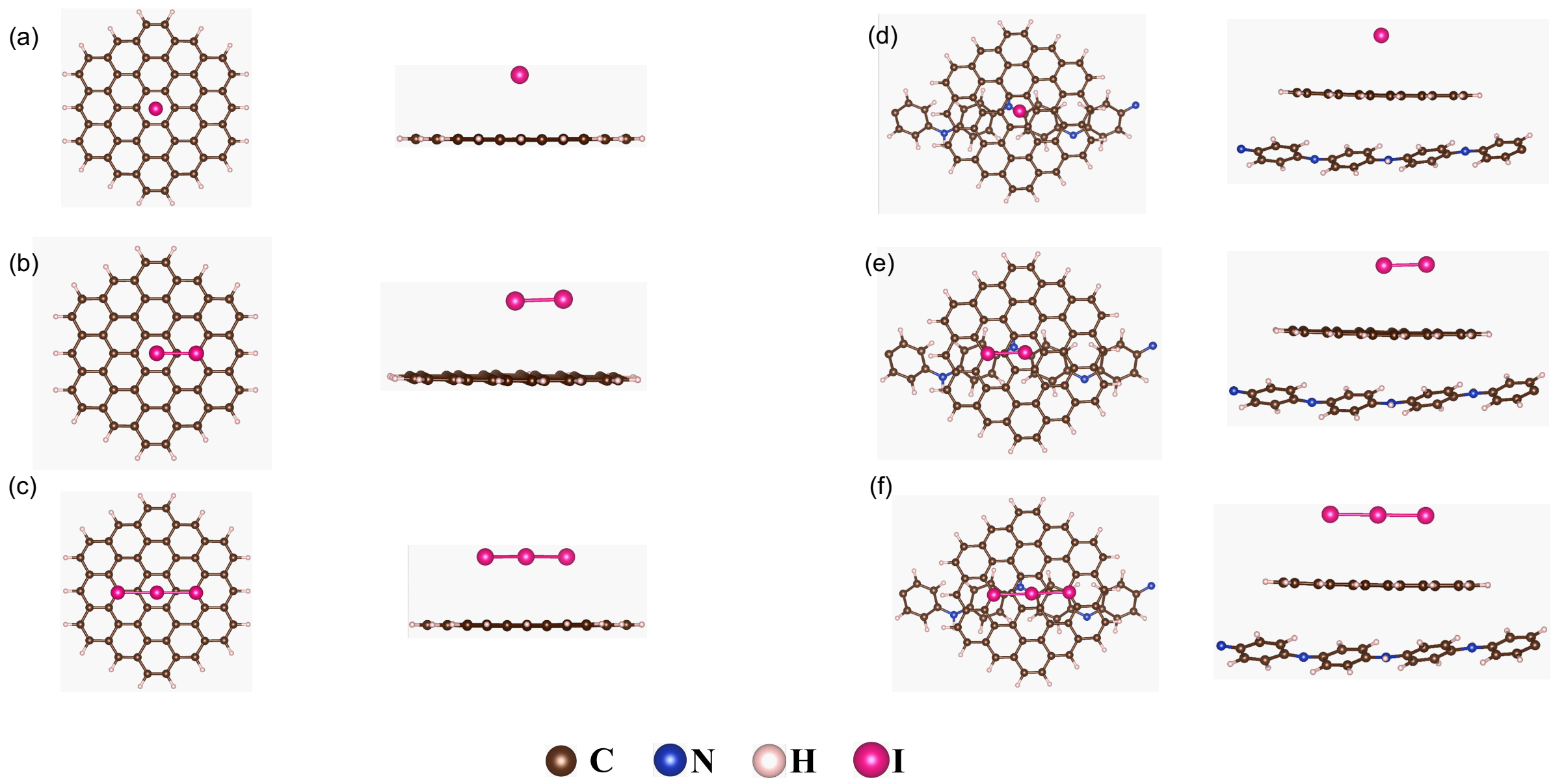

Figure S3. *Optimised geometry of ionic species I$^-$, I$_2$, and I$_3^-$ adsorbed on AC (a-c), and on AC-PANI (d-f).*

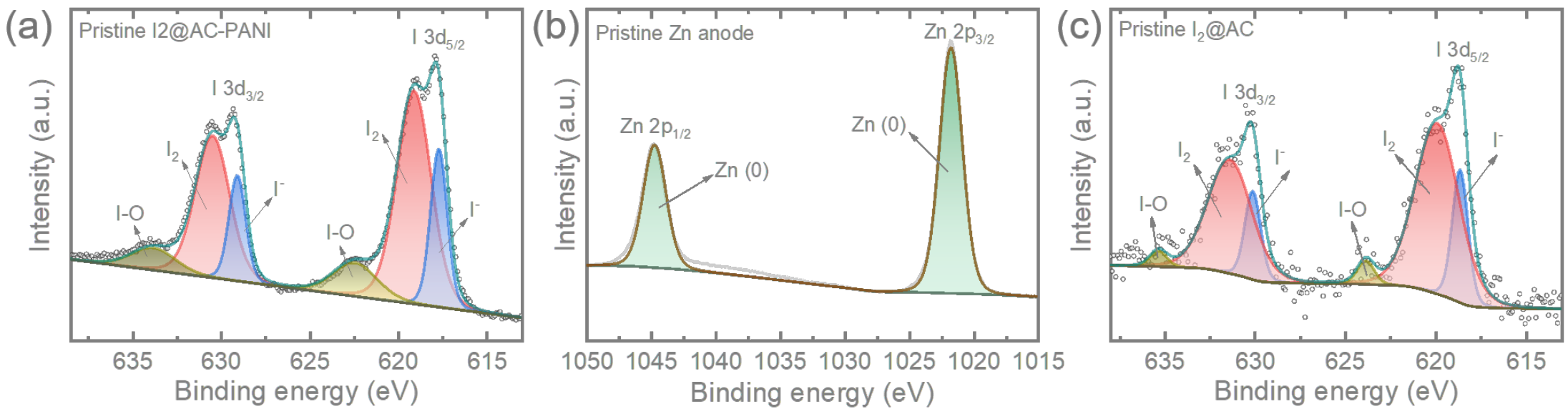

Figure S4. *XPS spectra of pristine (a) I_2@AC-PANI cathode I 3d, (b) Zn anode Zn 2p, and (c) I_2@AC cathode I 3d.*

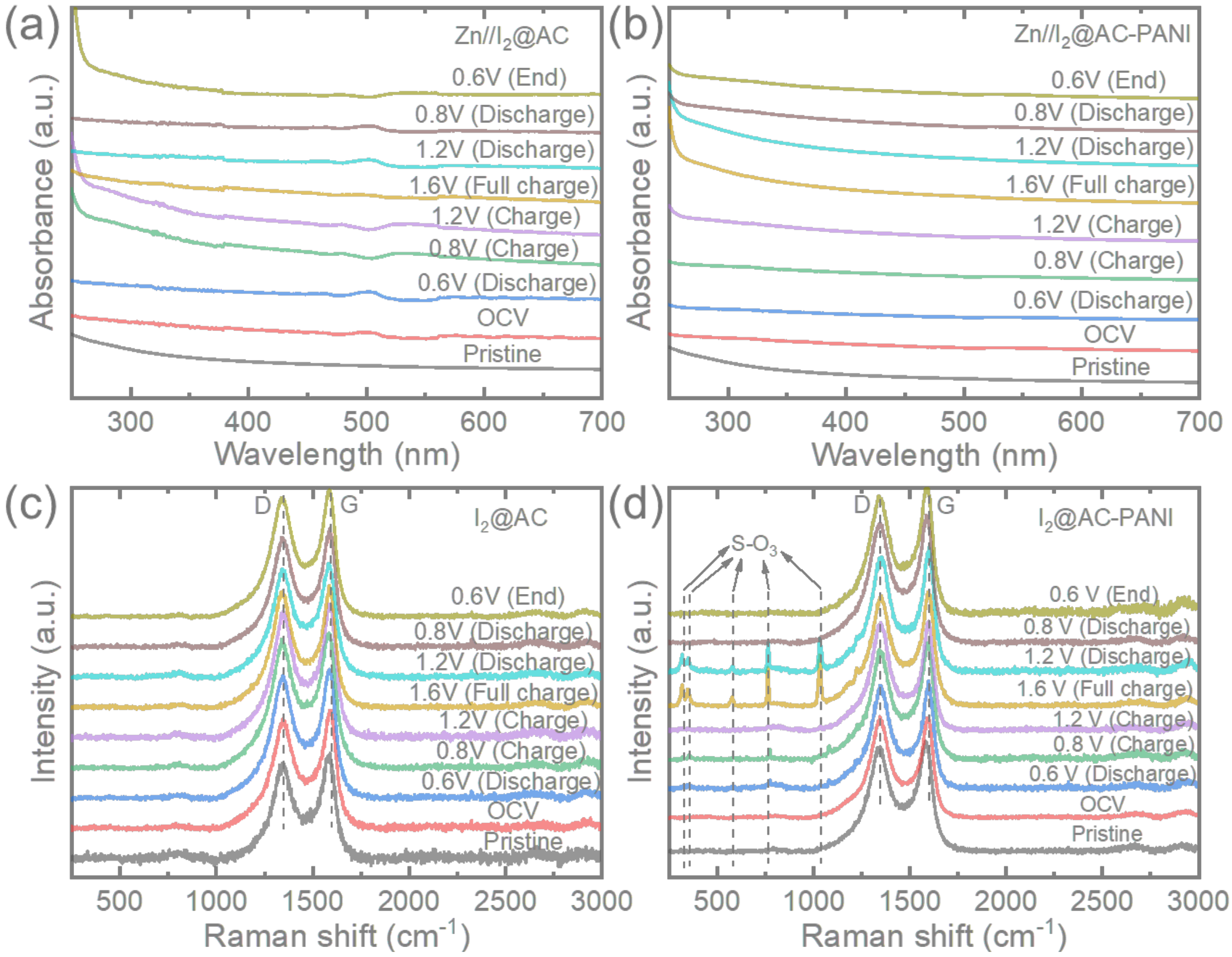

Figure S5. *Ex-situ UV-Vis spectra of 3M $Zn(CF_3SO_3)_2$ electrolyte of (a) Zn//I_2@AC and (b) Zn//I_2@AC-PANI cells. Ex situ Raman spectra of I_2@AC and I_2@AC-PANI cathodes at different states of charge.*

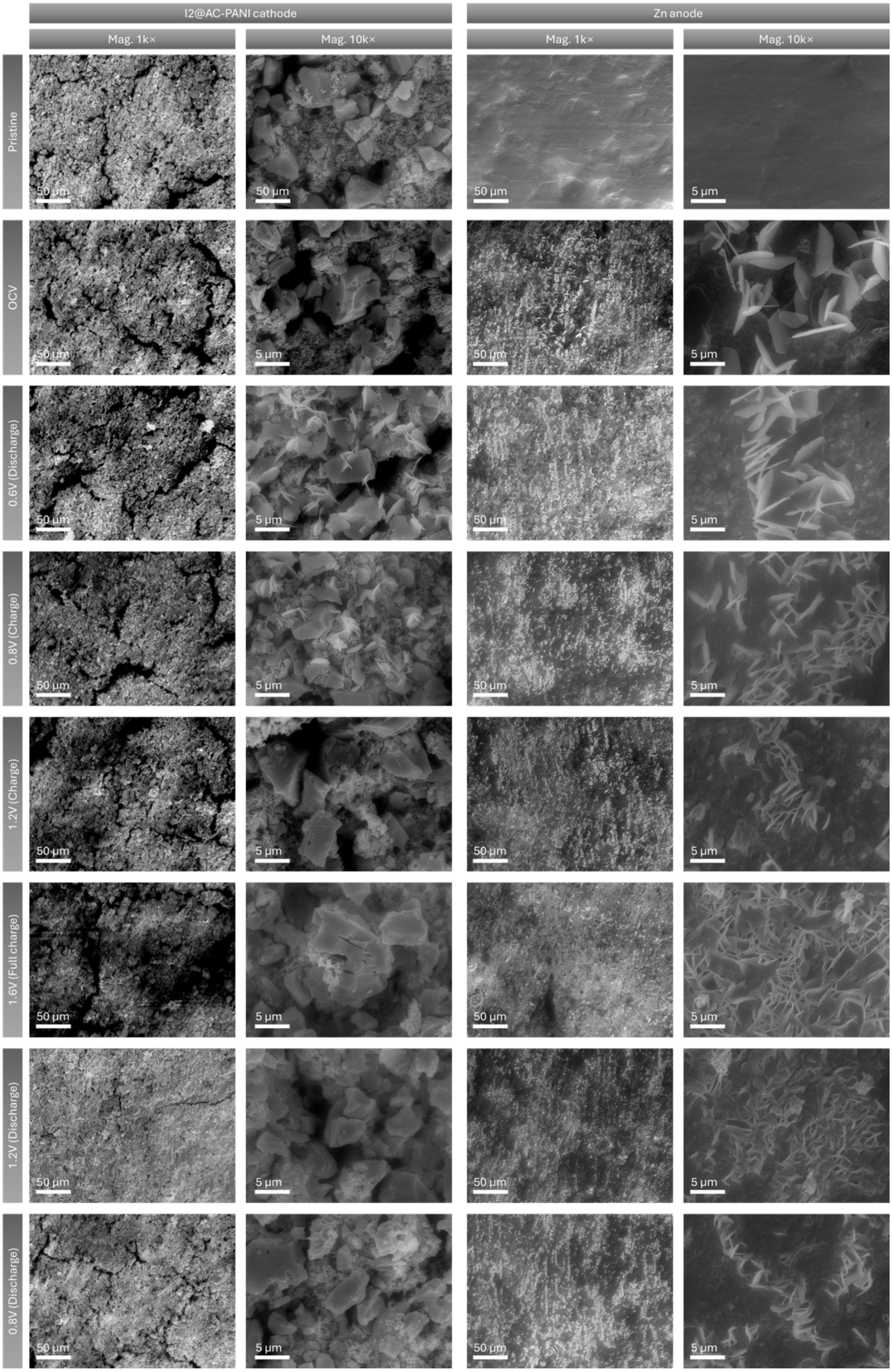

***Figure S6**. Ex-situ SEM images of I_2@AC-PANI cathode and Zn anode at different selected states of charge points.*

REFERENCES

1. Bartolotti, L. J. & Flurchick, K. An introduction to density functional theory. *Rev. Comput. Chem.* 187–216 (1996).

2. Argaman, N. & Makov, G. Density functional theory: An introduction. *Am. J. Phys.* **68**, 69–79 (2000).

3. Harrison, N. M. An introduction to density functional theory. *Nato Sci. Ser. Sub Ser. III Comput. Syst. Sci.* **187**, 45–70 (2003).

4. Kresse, G. & Furthmüller, J. Efficiency of ab-initio total energy calculations for metals and semiconductors using a plane-wave basis set. *Comput. Mater. Sci.* **6**, 15–50 (1996).

5. Kresse, G. & Furthmüller, J. Efficient iterative schemes for ab initio total-energy calculations using a plane-wave basis set. *Phys. Rev. B* **54**, 11169 (1996).

6. Perdew, J. P., Burke, K. & Ernzerhof, M. Generalized gradient approximation made simple. *Phys. Rev. Lett.* **77**, 3865 (1996).

7. Blöchl, P. E. Projector augmented-wave method. *Phys. Rev. B* **50**, 17953 (1994).

8. Grimme, S., Antony, J., Ehrlich, S. & Krieg, H. A consistent and accurate ab initio parametrization of density functional dispersion correction (DFT-D) for the 94 elements H-Pu. *J. Chem. Phys.* **132**, (2010).

9. Naresh, N. *et al.* 3D Engineered Dual-Redox Zinc-Iodine Microbatteries for Intrinsically Safe on-Chip Energy Storage. *Adv. Sci.* e75453 (2026).
